# Role of laser therapy in enhancing chemotherapy efficiency in breast cancer: low level laser therapy, photochemotherapy, and photodynamic therapy as promising treatments

**DOI:** 10.1007/s10103-025-04771-7

**Published:** 2026-02-24

**Authors:** Aya E. Mohamed, Wafaa R. Mohamed, Mai A. Elhemely, Tarek Mohamed

**Affiliations:** 1 Laser Institute for Research and Applications (LIRA), Beni-Suef University, Banī Suwayf, Egypt; 2 Department of Pharmacology and Toxicology, Faculty of Pharmacy, Beni-Suef University, Banī Suwayf, Egypt; 3 Laser Institute for Research and Applications (LIRA), Beni-Suef University, Banī Suwayf, Egypt; 4 School of Medical Sciences, Faculty of Biology, Medicine and Health, University of Manchester, Manchester, United Kingdom

**Keywords:** Breast cancer, Chemotherapy, Low level laser therapy, Cytochrome c, Photochemotherapy, Photodynamic therapy

## Abstract

Breast cancer is one of the most prevalent and biologically diverse malignancies in women worldwide, encompassing subtypes such as ductal carcinoma in situ (DCIS), lobular carcinoma, and triple-negative breast cancer (TNBC). These variants present complex therapeutic challenges. Chemotherapy remains a core treatment modality, particularly in aggressive or advanced stages, yet its systemic toxicity and lack of specificity limit its efficacy. In recent years, laser-based therapies have emerged as adjunctive strategies to enhance therapeutic precision. This review explores breast cancer classification, progression, and treatment, with an emphasis on chemotherapy, and critically examines the emerging role of laser technologies, including low-level laser therapy (LLLT), Photochemotherapy, and Photodynamic Therapy (PDT), as adjunctive or alternative therapeutic options. We highlight the potential of laser to modulate the tumor microenvironment, improve drug delivery, regulate mitochondrial function, and enhance apoptosis. PDT showed promise in activating localized cytotoxic effects while sparing surrounding tissues. However, heterogeneity in laser parameters and treatment protocols remains a significant barrier to clinical translation. This review underscores the translational potential of laser-assisted chemotherapy, identifies current gaps, and suggests future research directions for optimized treatment strategies in breast oncology.

## Highlights

Breast cancer subtypes pose significant diagnostic and therapeutic challenges.

Laser-based therapies are emerging as promising strategies in breast cancer treatment.

Photobiomodulation modulates tumor microenvironment and promotes tissue regeneration.

Photochemotherapy involves light interaction with chemotherapy, improving antitumor effect.

Photodynamic Therapy involves photosensitizer activation by laser to generate ROS.

## Introduction

Cancer is a group of diseases characterized by the uncontrolled proliferation of abnormal cells, which can invade surrounding tissues and spread through the blood and lymphatic systems [1, 2]. It remains a major global health concern. In 2022, lung cancer accounted for 12.4% of all newly diagnosed cancer cases, followed by breast cancer (11.6%), colorectal, prostate, and stomach cancers [3]. Cancer-related mortality rates indicate that lung cancer remains the leading cause of cancer deaths, followed by colorectal, liver, breast, and stomach cancers [4–6]. Several lifestyle and environmental factors, including tobacco use, alcohol consumption, poor diet, physical inactivity, and exposure to carcinogens, significantly contribute to cancer development. Additionally, chronic infections such as Helicobacter pylori, human papillomavirus (HPV), and hepatitis B and C are major risk factors, particularly in low- and middle-income countries [7, 8]. Among all cancer types, breast cancer remains the most prevalent malignancy among women worldwide and represents a significant public health challenge. Despite advances in screening and treatment, mortality remains high due to tumor heterogeneity, metastasis, and the development of resistance to conventional therapies [9]. Chemotherapy remains a cornerstone in breast cancer management; however, its non-selective cytotoxicity, severe systemic toxicity, and narrow therapeutic index highlight the urgent need for more safe and more targeted approaches [10]. In this context, light-based therapies—such as low-level laser therapy (LLLT), photochemotherapy, and photodynamic therapy (PDT)—have gained attention as innovative strategies for modulating cellular metabolism, enhancing drug response, and minimizing collateral tissue damage [11, 12]. Recent advances in photomedicine and ultrashort-pulse laser technology have shown promise in selectively inducing apoptosis in tumor cells, improving local immune activation, and overcoming multidrug resistance [13, 14]. Therefore, this review is structured to first provide an overview of chemotherapy as the conventional backbone of treatment, followed by a discussion of emerging laser-based phototherapeutic strategies. Finally, we compare their respective strengths and limitations, highlight current research gaps, and propose future perspectives for integrating chemotherapy with laser-based approaches in clinical oncology.

## Breast cancer

Breast cancer is one of the most prevalent malignancies worldwide. While it affects both men and women, its incidence is significantly higher in women [15]. The breast consists of lobules (milk-producing glands), ducts (which transport milk to the nipple), and connective tissues. Most breast cancers arise in the ducts (ductal carcinoma), while others develop in the lobules (lobular carcinoma). Several rare subtypes of breast cancer exist, each with distinct pathological and clinical characteristics. Invasive lobular carcinoma (ILC) originates in the lobules and is known for its diffuse growth pattern. Inflammatory breast cancer (IBC) is an aggressive form that presents redness, swelling, and warmth, often mimicking an infection [16]. Paget’s disease of the nipple arises in the milk ducts and extends to the nipple and areola, causing eczema-like changes [17]. Phyllodes tumors, which develop in the breast’s connective tissue, can be benign, borderline, or malignant [18]. Additionally, angiosarcoma, a rare and aggressive cancer, originates from endothelial cells lining blood or lymphatic vessels [19].

According to GLOBOCAN 2022, approximately 2.31 million new breast cancer cases and 669,846 related deaths were reported globally, highlighting its persistent global health burden [20] [16]. Mortality rates remain disproportionately higher in developing countries due to limited early detection and treatment accessibility [21].

Breast cancer etiology is multifactorial, influenced by lifestyle, hormonal, genetic, and environmental factors. Lifestyle factors such as obesity, physical inactivity, and alcohol consumption are strongly associated with increased risk, especially in postmenopausal women, through mechanisms involving chronic inflammation, hormonal imbalance, and metabolic dysfunction [22, 23]. Hormonal influences—such as prolonged use of oral contraceptives, hormone replacement therapy, and delayed pregnancy—contribute to excessive estrogen and progesterone exposure, promoting abnormal cellular proliferation [24]. In addition to hormonal and lifestyle factors, genetic predisposition is a major determinant of breast cancer susceptibility. Mutations in key tumor suppressor genes, including BRCA1, BRCA2, TP53, ataxia-telangiectasia (ATM), checkpoint kinase 2 (CHEK2), RAD51 paralog C, and BRCA1-associated RING domain 1 (BARD1), significantly heighten the risk of developing breast cancer due to their roles in DNA repair and cell cycle regulation. Individuals with BRCA1/2 mutations, in particular, face a substantially higher lifetime risk, making genetic screening an essential tool for early detection and prevention [25, 26]. Environmental and familial factors also contribute to breast cancer incidence. Exposure to ionizing radiation and carcinogenic chemicals has been implicated in breast cancer development by inducing DNA damage and genomic instability [27]. Additionally, a family history of breast cancer, particularly in first-degree relatives, is a well-established risk factor, indicating a possible hereditary component beyond known genetic mutations [28]. Histological and molecular classifications guide diagnosis, prognosis, and therapeutic decisions.

### Histological classification

The histological classification of breast cancer delineates distinct types based on cancer cells’ microscopic appearance and behavior. These types include ductal carcinoma in situ (DCIS), invasive ductal carcinoma (IDC), Lobular carcinoma in situ (LCIS), and ILC [29]. DCIS is a non-invasive type where abnormal cells are confined within the milk ducts, representing the earliest form of breast cancer with significant potential for progression if untreated [30]. In contrast, IDC, the most prevalent type, arises in the milk ducts and invades the surrounding breast tissue [31]. LCIS, found in the lobules, is not a true cancer but a marker indicating an elevated risk of developing invasive breast cancer in either breast. ILC, originating in the lobules, invades surrounding tissue and is often characterized by a distinct single-file pattern of spread, which helps differentiate it from IDC [32].

### Molecular classification

**Fig. 1:**
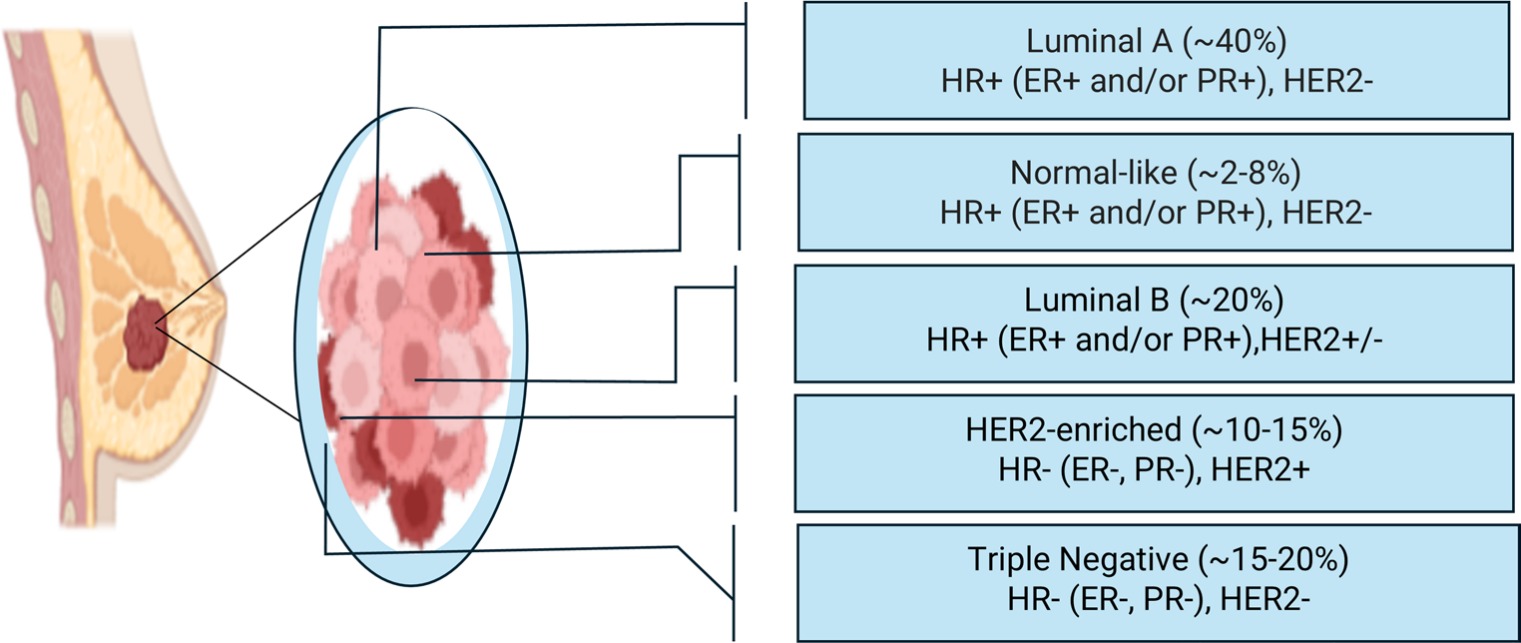
Breast cancer types and their prevalence. HR: Hormone, ER: Estrogen, PR: Progesterone, HER2: Human epidermal growth factor receptor 2

The molecular classification of breast cancer categorizes tumors based on the genetic characteristics of cancer cells. The molecular subtypes include luminal A, luminal B, human epidermal growth factor receptor 2 (HER2)-enriched, and triple-negative/basal-like [33] Fig. 1. Luminal A cancers are hormone receptor-positive, meaning they are estrogen receptor-positive (ER+) and/or progesterone receptor-positive (PR+), and HER2-negative. This subtype is associated with a relatively good prognosis and lower proliferation rates. Luminal B cancers are also hormone receptor-positive but can be either HER2-positive or HER2-negative and have a higher proliferation rate, leading to a somewhat poorer prognosis compared to Luminal A [34]. HER2-enriched cancers are hormone receptor-negative (ER- and PR-) and HER2-positive, typically exhibiting more aggressive behavior but responding well to targeted HER2 therapies [35]. Triple-negative/basal-like breast cancers (TNBC) lack estrogen receptors, progesterone receptors, and HER2. They are often associated with a poorer prognosis due to their aggressive nature and lack of targeted hormonal or HER2-directed treatments [36].Overall, understanding the diverse biological, molecular, and etiological aspects of breast cancer is essential for guiding precision therapy and improving clinical outcomes. The following sections explore conventional chemotherapy as the cornerstone of breast cancer management, followed by the emerging potential of laser-based phototherapies and their integration into modern oncologic practice.

## Therapeutic approaches for breast cancer

Treatment strategies for breast cancer generally involve a combination of surgery, chemotherapy, and radiation therapy, with ongoing research focused on developing new targeted therapies and immunotherapies [37]. Surgery is often the first line of treatment for breast cancer and typically involves either breast-conserving surgery (lumpectomy) or surgical breast removal (mastectomy) to remove the tumor [38, 39]. Radiation therapy is often recommended after surgery to minimize the risk of tumor recurrence, particularly in patients who undergo breast-conserving surgery. In certain cases, neoadjuvant radiation therapy may be administered before surgery to shrink large or locally advanced tumors, thereby facilitating surgical removal and improving overall treatment outcomes [40–42]. Targeted therapies may be effective against other molecular pathways involved in breast cancer, as TNBC lacks specific targets like hormone receptors or HER2. For example, Poly(ADP-Ribose) Polymerase 1 (PARP) inhibitors may be used in TNBC cases with BRCA mutations [43, 44]. Immunotherapy, particularly immune checkpoint inhibitors like pembrolizumab or atezolizumab, has shown promise in treating a subset of TNBC cases with high levels of TILs [45]. Chemotherapy is a standard treatment option for breast cancer, given its aggressive nature. Anthracycline-based chemotherapy, taxane-based chemotherapy, platinum compounds, or a combination of them are commonly used regimens [46].

### Chemotherapy

Chemotherapy is a cornerstone of treatment for breast cancer by targeting and killing rapidly dividing cancer cells. Chemotherapy is employed in various settings: neoadjuvant, adjuvant, and for metastatic disease [47]. Neoadjuvant Chemotherapy helps shrink tumors, making them easier to remove surgically. It also provides an opportunity to evaluate the tumor’s response to treatment [48]. Adjuvant Chemotherapy aims to destroy any remaining cancer cells and reduce the risk of recurrence. The major classes of agentsinclude anthracyclines, taxanes, and platinum-based compounds, each with distinct mechanisms and clinical applications [49, 50]. Among these, anthracyclines such as doxorubicin and epirubicin represent standard therapeutic options, often used in combination with taxanes [51]. Anthracyclines exert their effect by intercalating with DNA, leading to destabilization and subsequent degradation via DNA repair cascades, which can be beneficial in treating breast cancer [47]. Studies suggest that anthracyclines act directly by targeting cancerous tissues and indirectly via activating the immune system by stimulating CD8 + T cells [52]. Clinical studies confirm that anthracyclines improve response rates and extend survival, especially when used as part of neoadjuvant regimens [53].

Patients with normal BRCA1/2 genes (wild type) who have not previously been treated with anthracyclines or taxanes during earlier therapies, such as neoadjuvant or adjuvant treatments, are usually prescribed chemotherapy regimens based on these drugs [54]. While studies suggest that reusing anthracyclines or taxanes (a process known as rechallenging) can be effective in some cases, many doctors are cautious about re-administering anthracyclines. This caution stems from the risk of heart damage, as the cumulative use of anthracyclines increases the chance of cardiac toxicity over time [54–56]. Although anthracycline-based therapies can yield high initial response rates, their clinical use is limited due to increased recurrence and reduced overall survival. Moreover, these treatments are linked to severe acute toxicities, such as irreversible cardiotoxicity, myelotoxicity, alopecia, nausea, and vomiting, which significantly restrict their broader application [57].

Taxanes, another essential chemotherapeutic group, exert their antitumor effect by stabilizing microtubules, thereby disrupting mitotic spindle formation and cell division, and also inhibiting angiogenesis [58]. Studies indicate that taxanes, used alone or in combination with anthracyclines, offer benefits in TNBC, achieving pathological complete response rates of at least 40% [59, 60]. Currently, three taxanes, docetaxel, paclitaxel, and cabazitaxel, are approved for clinical use [61].

Docetaxel, developed in the 1980 s, remains one of the most effective agents for cancer therapy. However, its use can be limited by the emergence of antibiotic resistance, likely due to its impact on the body’s normal microbial flora [62]. Paclitaxel is highly valued for its specific, reversible, and saturable binding to microtubules, where it disrupts mitosis and induces tumor cell death. It has shown strong efficacy against a wide range of cancers, including breast, ovarian, prostate, lung, and others [63]. Despite its potency, issues such as resistance development and toxicity to healthy tissues highlight the importance of using it in combination regimens for optimal breast cancer management [64]. Cabazitaxel, a more recent addition to the taxane family, has shown strong performance in both in vitro and in vivo models, especially against tumors resistant to docetaxel and paclitaxel [65]. Its enhanced activity is linked to quicker cellular uptake and prolonged retention in cancer cells [66].

Platinum-based agents such as cisplatin and carboplatin have also been evaluated extensively in neoadjuvant and adjuvant breast cancer therapy [67, 68]. Platinum-based neoadjuvant chemotherapy shows a notable increase in pathological complete response rates among breast cancer patients, yet it comes with the downside of heightened hematological toxicities. Therefore, it remains a potential option for breast cancer treatment [69].

Cisplatin is a widely used anticancer drug for treating several solid tumors, including germ cell tumors, sarcomas, and lymphomas. Its effectiveness is linked to its cis-configuration, which features a central platinum atom coordinated with two stable amine ligands and two easily replaceable chloride ligands [70, 71]. However, breast cancer patients often experience cancer recurrence and metastasis after receiving cisplatin or carboplatin treatment due to drug resistance. In breast cancer treatment, cisplatin or carboplatin is usually combined with other medications such as paclitaxel, and it is frequently used with third-generation cytotoxic drugs such as vinca alkaloids, taxanes, or gemcitabine to treat a range of malignancies [72].

Its chemical structure is a central platinum atom bonded to two inert amine and two labile chlorine ligands. Upon entering cells through passive diffusion, cisplatin undergoes hydrolysis, where the chlorine ligands are replaced by water molecules, rendering the compound highly reactive and able to bind to DNA [26]. The primary mode of cisplatin’s interaction with DNA is the formation of cisplatin-DNA adducts, predominantly intrastrand cross-links (90–95%), which occur at purine base N7 sites [73]. These DNA adducts distort the DNA structure, preventing proper transcription and replication, and trigger cell signaling pathways that ultimately lead to apoptosis [74].

Non-DNA binding forms of cisplatin also contribute to its anticancer effects by generating reactive oxygen species (ROS), which can lead to oxidative stress [75]. This imbalance in ROS regulation can overwhelm cellular defense mechanisms, such as CAT, SOD, and GSH, resulting in cell damage and initiating a cascade of processes that lead to mitochondrial dysfunction and apoptosis [76]. Cisplatin can activate the intrinsic apoptotic pathway by inducing mitochondrial rupture, which releases pro-caspase-9 and cytochrome C, triggering the formation of an apoptotic complex [77]. This complex activates caspase-9, which then initiates caspase-3, −6, and − 7, culminating in cell death. Additionally, cisplatin can induce apoptosis through the extrinsic pathway, involving the Fas receptor and its ligand, leading to caspase activation [78].

Despite its effectiveness, platinum drugs are limited by significant toxicity and the development of drug resistance. Cisplatin’s toxicity affects multiple organs, including nephrotoxicity, hepatotoxicity, neurotoxicity, cardiotoxicity, ototoxicity, and even the testicles. These adverse effects restrict the dose and frequency at which cisplatin can be safely administered [79]. Efforts to overcome platinum drug-induced organ toxicity while preserving its anticancer efficacy are ongoing. Several studies have explored strategies to protect against these side effects, such as using protective agents or modifying treatment regimens, without compromising the anticancer effect. These include the use of antioxidants [80], Specific inhibitors of mitochondrial damage [81], Novel delivery methods aimed at reducing platinum drug exposure to non-cancerous tissues [82], and photo-biomodulation therapy [83]. Overall, chemotherapy remains a fundamental therapeutic approach for breast cancer, yet its clinical limitations; especially drug resistance and systemic toxicity highlight the urgent need for innovative, targeted, and less invasive strategies such as phototherapy and laser-based modalities.

## Light-based therapy

Phototherapy refers to the therapeutic application of light at specific wavelengths [84]. Its origins trace back to ancient civilizations such as Egypt, India, and Greece, where sunlight was employed to manage conditions including vitiligo, rickets, and skin disorders. The Greeks practiced heliosis through sun and sand baths, while the Egyptians used colored crystals to channel sunlight for healing, reflecting early recognition of light as a restorative force [85]. In classical medicine, heliotherapy was formally practiced; Herodotus highlighted sunlight in health restoration, and in the 19th century, physicians such as Cauvin promoted its curative role in scurvy, rickets, and rheumatism [86].

Niels Finsen established the scientific foundation of modern phototherapy in the late 19th century. He demonstrated that red light could prevent suppuration in smallpox lesions and developed carbon arc phototherapy for lupus vulgaris, achievements that earned him the Nobel Prize in 1903. Finsen’s work led to the establishment of phototherapy institutes across Europe and extended clinical applications to conditions such as tuberculosis, with lasting relevance in modern medicine, for example, in neonatal jaundice [87].

### Laser therapy

Lasers represent a modern and precise evolution of light therapy. The principle of stimulated emission was first introduced by Einstein in 1917, eventually leading to the invention of the first functional ruby laser by Theodore Maiman in 1960 [88, 89]. Over subsequent decades, several types of lasers were developed, including the helium-neon, Nd: YAG, CO₂, and argon ion lasers, broadening medical and industrial applications [90].

Lasers differ fundamentally from conventional light sources due to their properties: high directionality, monochromaticity, and coherence. These characteristics make them particularly effective in medical applications [91]. The wavelength, defined as the distance between successive peaks of a light wave, is typically expressed in nanometers (nm). Laser power output is measured in milliwatts (mW) or watts (W), while power density or irradiance is quantified as watts per square centimeter (W/cm²) or milliwatts per square centimeter (mW/cm²), accounting for both the total power and the illuminated surface area [92]. Lasers can operate in two main modes: continuous wave (CW) and pulsed wave. CW lasers maintain constant light emission, requiring stable output power, whereas pulsed lasers emit short bursts of light separated by intervals, with parameters described in terms of peak power, average power, and energy per pulse [93]. The biological impact of laser irradiation depends critically on these physical parameters. For instance, longer wavelengths penetrate deeper into tissues, while variations in power density and emission mode determine whether the dominant effect is photothermal, photomechanical, or photochemical [94, 95]. These fundamental optical and physical characteristics form the basis for diverse biomedical applications, particularly the development of LLLT, where carefully optimized parameters are used to stimulate cellular responses without causing tissue damage.

### Low-level laser therapy (LLLT)

LLLT is a non-thermal light-based treatment that modulates biological processes, first introduced by Mester et al. in 1967. It typically operates with power outputs of 0.001–0.1 W, wavelengths between 300 and 10,600 nm, pulse rates of 0–5000 Hz, intensities of 0.01–10 W/cm², and doses of 0.01–100 J/cm² [96]. Within this range, the therapeutic wavelengths of 600–1100 nm fall into the “optical window” which is a spectral region where light penetration through biological tissues is maximized due to minimal absorption by hemoglobin and water. In this window, wavelengths of 600–700 nm are optimal for targeting superficial tissues, whereas 780–950 nm allow deeper tissue penetration [97–99]. Its biological efficacy depends on optimizing parameters such as wavelength, fluence, power density, and emission mode (continuous or pulsed) according to the target tissue and therapeutic goal [100–102]. LLLT has demonstrated significant potential in promoting wound healing, tissue regeneration, reducing inflammation, and modulating pain [103]. Mechanistically, the red and near-infrared light range (600–1100 nm) is absorbed by CCO, a mitochondrial chromophore that facilitates electron transport and enhances cellular bioenergetics [104].

**Fig. 2:**
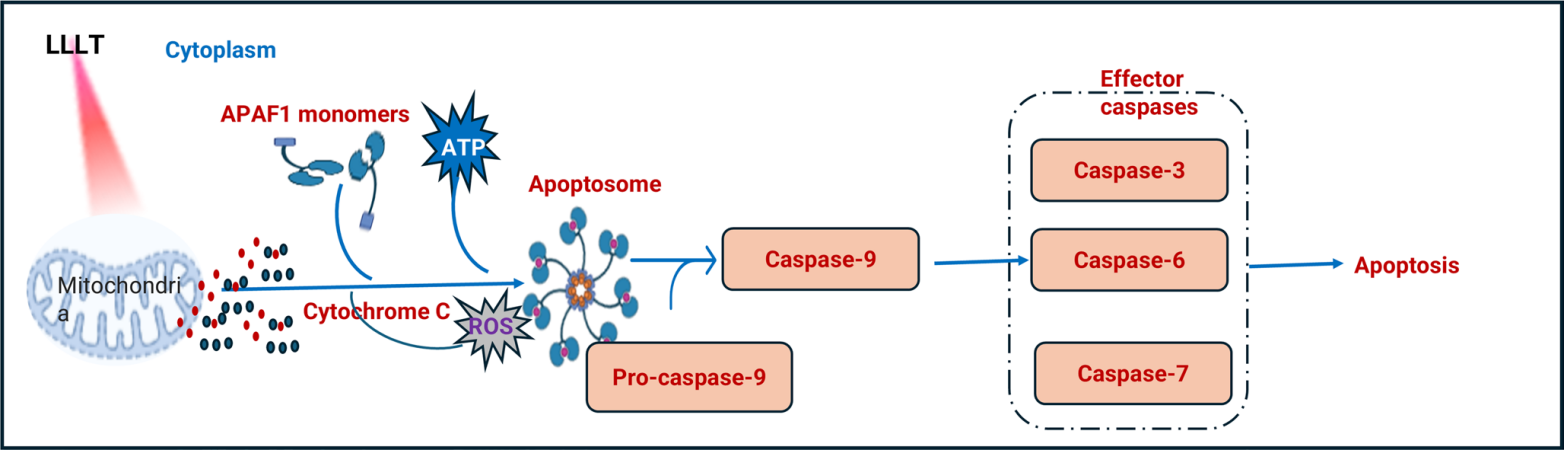
Mechanistic pathway for cellular response to LLLT LLLT: Low-level laser therapy, ROS: Reactive oxygen species, APAF1: Apoptotic protease-activating factor 1, ATP: Adenosine triphosphate

Mechanistically, the red and near-infrared light range (600–1100 nm) is absorbed by CCO, a mitochondrial chromophore that facilitates electron transport and enhances cellular bioenergetics [104]. CCO comprises several subunits containing heme (a, a3) and copper (CuA, CuB) that enable oxygen reduction and ATP synthesis [105]. Upon light absorption, CCO activity increases, leading to enhanced ATP production, modulation of ROS production, and mitochondrial outer membrane permeabilization [106]. This facilitates the release of cytochrome c into the cytosol, where it is associated with apoptotic protease-activating factor-1 (Apaf-1) and dATP to form the apoptosome. The apoptosome activates procaspase-9, triggering a caspase cascade that culminates in programmed cell death via executioner caspase-3 Fig. 2 [107], and regulation of nitric oxide (NO) levels [108]. The produced ATP activates key intracellular pathways, including MAPK and growth factor signaling (FGF2, EGF, NGF), and regulates ionic transporters such as Na⁺/K⁺ ATPase and calcium pumps, as well as cyclic AMP (cAMP) [109]. Studies have shown that exposure to 810 nm light enhances neurite outgrowth via ATP-mediated P2Y receptor activation, suggesting a role for G protein-coupled receptors in photo-biomodulation [110–113]. LLLT also modulates nitric oxide synthase (NOS) activity, affecting NO levels in a tissue-dependent manner, and can either stimulate or inhibit biological functions depending on the dose and energy level used [114, 115]. Previously, Al-Watban and Andres (2012) demonstrated a dose-dependent response in various cell lines, including normal cells such as Chinese hamster ovary cells and human skin fibroblasts, malignant cells (RIF-1 and EMT-6), and embryonic cells (3T3 and CCL-226 mouse embryonic fibroblasts), where lower doses induced biostimulatory effects such as proliferation and differentiation, while higher doses exerted bio-inhibitory actions capable of suppressing cancer cells. The optimal stimulatory dose for LLLT at 632.8 nm was found to be approximately 180 mJ/cm², while inhibitory effects were achieved at 420–600 mJ/cm² [116]. These findings collectively highlight the dual nature of LLLT, where controlled modulation of energy dose and wavelength determines whether the response is regenerative or suppressive, offering a promising basis for therapeutic application in cancer research [117]. Given these multifaceted biological effects, LLLT has attracted increasing attention as a complementary or alternative approach in oncology, particularly for breast cancer, where it may enhance therapeutic efficacy, reduce chemotherapeutic side effects [118], and selectively modulate cancer cell viability depending on the applied dose and wavelength. Several experimental and clinical studies have further demonstrated that the biological effects of LLLT rely strongly on the wavelength, energy dose, and exposure duration. For instance, Laura et al. reported that photo-switchable inhibitors targeting protein–protein interactions could be activated using 380 nm light, effectively suppressing activity in living cells, including HeLa, HEK293, and MCF7 lines [119]. In another investigation, MCF7 and MB-435 breast cancer cells were exposed in vitro to commonly used laser wavelengths (780 and 830 nm), and it was found that laser doses up to 5 J/cm² did not enhance cell proliferation [120]. Clinically, an 805 nm laser has been utilized for ablation of small breast tumors, depending on the specific ablation approach [121]. A study investigating 734 nm exposure on different cell types demonstrated that daily irradiation for 20 min over six days selectively induced senescence in MCF7 breast cancer cells, without affecting non-cancerous MCF10A breast cells or IMR-90 fibroblasts. This selective response was associated with mitochondrial dysfunction, characterized by elevated ROS levels and altered membrane potential (*p* < 0.05), while mitochondrial Ca²⁺ levels remained unchanged [122]. More recently, Ibrahim et al. (2024) demonstrated that the effect of LLLT on breast cancer cells (MCF-7) varies according to wavelength and dose, with near-infrared laser irradiation (780 nm, 25 mW for 900 s) producing the highest cytotoxicity and reducing cell viability to 32.53%. Collectively, these findings highlight the potential of LLLT as a wavelength- and dose-dependent therapeutic tool with selective anticancer properties [123]. These observations collectively emphasize that LLLT exerts wavelength- and dose-dependent biological effects that can be harnessed not only for direct modulation of cancer cell behavior but also for activating other light-responsive therapeutic strategies. Therefore, several LLLT-activated modalities, including photochemotherapy, PDT, have been developed to enhance the precision and efficacy of breast cancer treatment.

## LLLT-Activated therapeutic modalities in breast cancer treatment

### Photochemotherapy

**Fig. 3:**
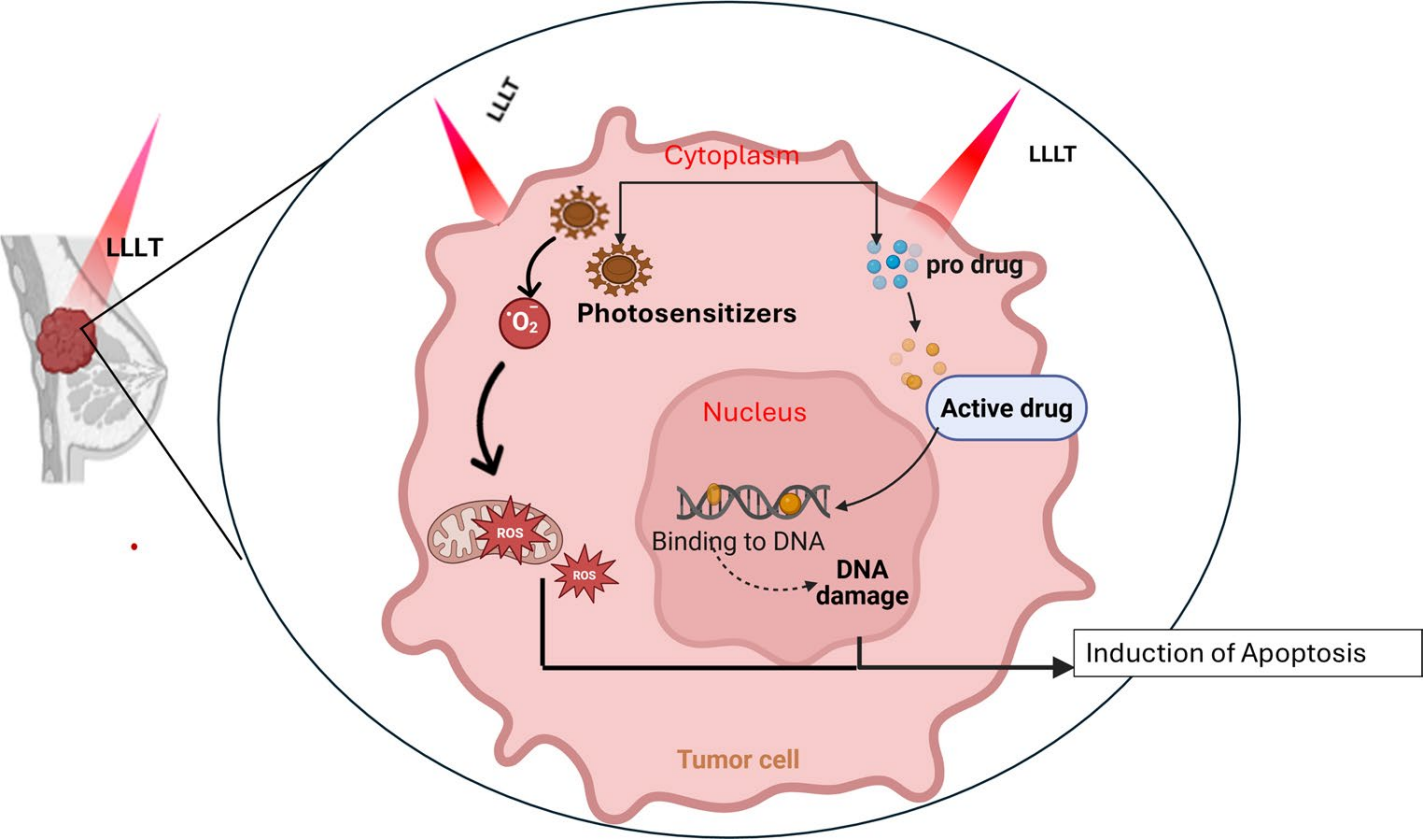
Mechanistic pathway for laser-induced apoptosis in photodynamic therapy and photochemotherapy LLLT: Low-level laser therapy, ROS: Reactive oxygen species

Photochemotherapy is a light-activated cancer treatment that combines the photochemical and photophysical properties of chemotherapy to enhance its antitumor effects [124]. This approach utilizes light-sensitive prodrugs that remain pharmacologically inactive until exposed to specific wavelengths of light, enabling precise spatial and temporal control of activation. Such targeted therapy is oxygen-independent and associated with fewer systemic side effects compared to conventional chemotherapy [125]. For effective cancer treatment, an ideal prodrug should be stable under normal physiological conditions, selectively accumulate in tumor cells, possess an adequate plasma half-life to reach the tumor site, and rapidly eliminate toxic byproducts after activation [126]. Activation can occur through several light-driven mechanisms, including photo-reduction, photo-substitution, ligand photocleavage, and photo-switching Fig. 3. However, despite significant progress, the clinical translation of many light-activated drugs remains limited due to challenges in improving photocytotoxicity at longer wavelengths required for deeper tissue penetration [127].

A major advantage of photochemical activation, particularly in transition-metal complexes, lies in their ability to form electronically excited states under mild conditions, facilitating efficient energy transfer and bond activation [128]. When a molecule absorbs a photon, it transitions from its ground state to a short-lived excited singlet state, which may return to a lower energy level through fluorescence, internal conversion, or non-radiative relaxation. Alternatively, it can undergo intersystem crossing (ISC) to a more stable triplet state that relaxes via phosphorescence [129, 130]. These electronic transitions often promote chemical transformations such as isomerization, dissociation, redox, or substitution reactions, which are relevant in activating platinum-based chemotherapeutic agents such as cisplatin. Once photoactivated, the platinum complex binds covalently to DNA, forming cytotoxic adducts that inhibit replication and transcription [131]. Experimental evidence shows that cisplatin is photosensitive—light at 350 nm can trigger photosubstitution reactions [132], and exposure to sunlight or ultrasound accelerates its degradation in chlorine-containing media [133]. Furthermore, synchrotron radiation studies revealed that photoexcitation induces the emission of Auger electrons from cisplatin’s valence shell, leading to enhanced DNA double-strand breaks and delayed repair, thereby intensifying its cytotoxic potential [134–136]. Combined cisplatin–laser light therapy significantly increases lipid peroxidation and enzymatic activity, indicating a synergistic enhancement of cytotoxicity and supporting its potential as a combinatorial cancer treatment strategy [137].

Similarly, doxorubicin, another potent chemotherapeutic, exerts cytotoxicity primarily by intercalating into DNA base pairs, inhibiting topoisomerase II, and generating ROS that activate caspase-3 and p53-mediated apoptotic cascades [138]. When coupled with infrared laser irradiation, doxorubicin’s apoptotic potential is further enhanced through ROS-induced amplification and mitochondrial signaling, highlighting a promising synergistic mechanism for light-assisted chemotherapy [139]. A previous study revealed that 660 nm and 810 nm laser irradiation potentiated doxorubicin’s effects, creating favorable conditions for apoptosis, as evidenced by cytomorphological analysis [140]. Another study demonstrated that 630 nm and 980 nm reduced the viability of doxorubicin-resistant MDA-MB-231 cells, as indicated by decreased IC50 values. Recent investigations have expanded this concept through nanotechnology-based platforms that integrate photosensitizers and chemotherapeutic agents into a single construct. For instance, a ROS/GSH-responsive camptothecin-based nanoplatform demonstrated selective cytotoxicity toward MCF-7 and 4T1 breast cancer cells under NIR light activation, achieving significant tumor suppression with minimal systemic toxicity [141]. Likewise, an indocyanine green–camptothecin nanocomposite produced nearly 80% tumor regression in MDA-MB-231 xenografts following 808 nm irradiation [142]. Another study employing a 3D multicellular breast cancer model revealed that sequential photochemotherapy—applying chemotherapy followed by light exposure—yielded greater cytotoxicity compared to simultaneous or reversed protocols [143]. Collectively, these findings underscore photochemotherapy’s potential as a precise, minimally invasive strategy for breast cancer treatment. Nonetheless, its clinical adoption remains constrained by limited light penetration depth, challenges in localized light delivery, and risks of off-target phototoxicity. Future efforts should focus on optimizing activation wavelengths, engineering biocompatible nanocarriers for selective drug delivery, and developing advanced optical systems capable of deep-tissue illumination to realize the therapeutic promise of photochemotherapy in oncology fully. Building upon these principles, another LLLT-activated modality—PDT—employs light to activate photosensitizing agents in the presence of oxygen, generating cytotoxic reactive species that selectively destroy tumor cells while sparing normal tissue.

### Photodynamic therapy (PDT)

PDT represents another promising light-based therapeutic approach for cancer management. PDT is a minimally invasive strategy that relies on the concurrent action of three essential components: a photosensitizer (PS), light of a specific wavelength, and molecular oxygen [144]. Upon illumination, the PS absorbs photons and transitions from its ground singlet state (¹PS) to an excited singlet state (¹PS*), which may relax through fluorescence or undergo intersystem crossing to form a more stable triplet state (³PS*). This triplet state drives the photochemical reactions responsible for cytotoxic effects [145]. Two main mechanisms govern PDT activity: in the type I pathway, ³PS* transfers electrons or hydrogen atoms to surrounding biomolecules, forming free radicals and radical ions that subsequently react with molecular oxygen to produce ROS such as superoxide (O₂⁻•), hydroxyl radicals (•OH), and hydrogen peroxide (H₂O₂) [146], in the type II pathway, ³PS* transfers energy directly to ground-state molecular oxygen (³O₂), generating singlet oxygen (¹O₂), a highly reactive form of oxygen that induces oxidative cellular damage [147] Fig. 3. While both mechanisms contribute to cytotoxicity, type II reactions typically predominate, and their relative contribution depends on PS type, oxygen availability, and light parameters [148].

PDT achieves tumor destruction through three interrelated mechanisms: (1) direct tumor cell killing via ROS-mediated apoptosis or necrosis, (2) vascular damage leading to reduced oxygen and nutrient supply, and (3) stimulation of local inflammation and antitumor immune responses [149]. The therapeutic success of PDT depends on the PS localization, light fluence and fluence rate, tissue oxygenation, and drug–light interval [150]. In breast cancer, several experimental and clinical studies have validated PDT’s potential [151]. Effective tumor necrosis and regression were demonstrated at a fluence of 90 J/cm² in breast lesions smaller than 10 mm, while a Phase II clinical trial using motexafin lutetium (MLu, 4–5 mg/kg) combined with 730 nm laser irradiation (150 J/cm², 75 mW/cm²) achieved therapeutic responses in recurrent breast carcinoma [152, 153]. PDT has also been successfully applied in Paget’s disease of the breast [154, 155]. Photofrin-mediated PDT (630 nm) achieved a complete histologic response in 8 of 9 patients with chest wall recurrence [156]. Moreover, meta-tetra(hydroxyphenyl)chlorin (m-THPC) at 652 nm (5–10 J/cm², 20–25 mW/cm²) yielded a complete response in all patients with metastatic skin nodules and exhibited enhanced cytotoxicity in doxorubicin-resistant MCF-7/DXR cells, suggesting its utility in overcoming chemoresistance [156, 157]. Similarly, verteporfin (690 nm, 150 mW/cm²) demonstrated therapeutic benefits in invasive ductal carcinoma [158].

Recent technological advances have integrated femtosecond laser systems into PDT to enhance precision and control. Femtosecond lasers, characterized by ultrashort pulse durations, enable localized photochemical activation with minimal thermal injury [159]. Chen et al. reported that femtosecond laser irradiation of FePt nanoparticles effectively inhibited EMT-6 breast cancer cell proliferation through synergistic photothermal and photodynamic mechanisms [160]. Likewise, amino levulinate-mediated photosensitization using femtosecond laser exposure induced selective cytotoxicity in breast, skin, and bladder cancer cells, emphasizing the potential of ultrafast light sources to refine PDT and broaden its applicability across diverse tumor types [161]. However, despite encouraging progress, several translational challenges remain. The limited penetration depth of activating light restricts PDT efficacy in deep-seated breast tumors, while tumor hypoxia reduces singlet oxygen generation efficiency. Additionally, most existing evidence derives from in vitro and small-scale clinical studies, with few randomized phase II/III trials validating long-term survival or recurrence outcomes [162]. Current research trends focus on oxygen-generating nanoplatforms, femtosecond or interstitial laser delivery systems, and combined PDT–chemotherapy or PDT–photothermal approaches, all designed to enhance selectivity, reduce systemic toxicity, and overcome tumor resistance. These findings suggest that PDT and photochemotherapy represent complementary and promising modalities in breast cancer management, especially when coupled with targeted nanotechnology and controlled light activation.

A Summary of representative studies on light-based therapies for breast cancer is listed in Table 1.

**Table 1:** Summary of representative studies on light-based therapies for breast cancer

Study/Reference	Model/Cell Line or Subject	Wavelength/Dose	Type of Therapy	Key Findings/Mechanism	Outcome/Conclusion
[116]	CHO, human fibroblasts, RIF-1, EMT-6, 3T3, CCL-226	632.8 nm, 180–600 mJ/cm²	LLLT	Low doses induced proliferation; high doses inhibited cancer cells	Dose-dependent dual effect (stimulatory vs. inhibitory)
[119]	HeLa, HEK293, MCF7	380 nm	Photochemotherapy	Photo-switchable inhibitors suppressed protein–protein interactions	Light-controlled inhibition of cancer signaling
[120]	MCF7, MB-435	780 & 830 nm ≤ 5 J/cm²	LLLT	No significant increase in proliferation	Safe exposure range for LLLT
[122]	MCF7, MCF10A, IMR-90	734 nm, 20 min/day for 6 days	LLLT	Induced senescence in MCF7 cells, not normal cells	Selective mitochondrial dysfunction
[123]	MCF7	780 nm, 25 mW for 900 s	LLLT	Near-IR exposure caused 67% cell death	Wavelength- and dose-dependent cytotoxicity
[137]	LNCaP prostate cancer	350 nm	Photochemotherapy	Laser enhances DNA damage	Synergistic cytotoxicity with cisplatin
[140]	MDA-MB-231	630 & 980 nm	Photochemotherapy	Laser enhances ROS and apoptotic signaling	Improved efficacy of doxorubicin in resistant cells
[141]	MCF7, 4T1	NIR light	Photochemotherapy	Light-activated Camptothecin nanoplatform selective cytotoxicity	Strong tumor suppression and low toxicity
[142]	MDA-MB-231 xenografts	808 nm	Photochemotherapy	Enhanced photochemical activation	~ 80% tumor regression
[151]	Clinical breast lesions	90 J/cm²	PDT	Effective tumor destruction < 10 mm	Demonstrated clinical efficacy
[152]	Recurrent breast carcinoma	730 nm, 150 J/cm²	PDT	Light-activated Motexafin lutetium and achieved therapeutic response	Promising phase II results
[157]	MCF-7/DXR, skin metastases	652 nm, 5–10 J/cm²	PDT	Apoptosis in drug-resistant cells	m-THPC overcame chemoresistance
[158]	Invasive ductal carcinoma	690 nm, 150 mW/cm²	PDT	Selective phototoxicity	Validated clinical benefit
[160]	EMT-6 cells	Femtosecond laser (700 to 990 nm)	PDT/Photo thermos therapy combined	Femtosecond laser with FePt NPs enhanced ROS generation and cytotoxicity	Synergistic growth inhibition

## Clinical translation and regulatory status

Several light-based therapies have reached clinical application, while others remain in preclinical or investigational stages. PDT using porfimer sodium (Photofrin^®^) has been FDA-approved for the treatment of various solid tumors, including non-small-cell lung cancers, and has shown promising off-label results in breast cancer recurrence. Other photosensitizers, such as temoporfin and verteporfin are clinically approved in Europe and under evaluation for metastatic and locally recurrent breast cancers [163].

Conversely, LLLT and LLLT-assisted chemotherapeutic or photochemical approaches remain largely experimental, with ongoing clinical trials exploring their safety, dosimetry optimization, and long-term efficacy in breast cancer management. Similarly, femtosecond laser–based phototherapies and nanotechnology-assisted light activation platforms are still in early translational phases, showing encouraging preclinical data but lacking FDA approval at present.

Overall, while PDT represents the most clinically established modality, the integration of LLLT and ultrafast laser systems holds significant potential for the future of minimally invasive, precision-guided cancer therapy.

## Conclusion and perspectives

**Fig. 4:**
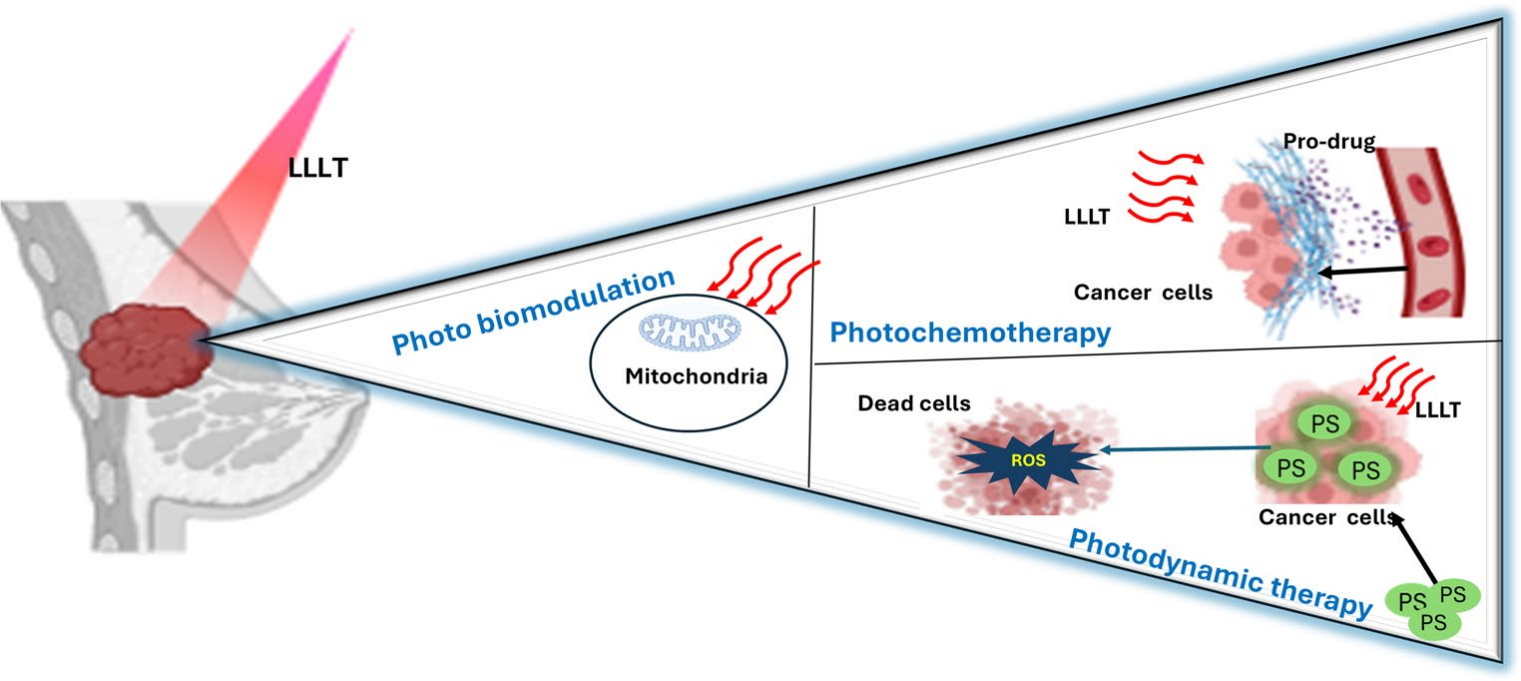
Schematic presentation of low-level laser-based therapies in breast cancer treatment PS: photosensitizers, APAF1: Apoptotic protease activating factor 1

Breast cancer continues to represent a major global health burden, with conventional treatments such as surgery, radiotherapy, and chemotherapy often limited by systemic toxicity, drug resistance, and poor selectivity. Recent progress in light-based therapies, particularly LLLT, PDT and photochemotherapy, has provided new avenues for precise and minimally invasive cancer management Fig. 4. These modalities harness the ability of specific light wavelengths to modulate mitochondrial and molecular signaling, inducing selective apoptosis, immune activation, and vascular remodeling while sparing surrounding healthy tissues.

LLLT-based interventions have shown potential in enhancing the therapeutic index of existing treatments by improving tumor selectivity. PDT combines a photosensitizer, oxygen, and light to produce cytotoxic singlet oxygen species, resulting in effective tumor destruction with minimal recurrence. Furthermore, the integration of nanotechnology and ultrafast femtosecond lasers has introduced new dimensions of precision, enabling controlled photochemical activation and spatiotemporal selectivity at the cellular and subcellular levels. Despite these promising outcomes, several limitations remain. Light penetration depth, variability in tissue oxygenation, non-standardized dosimetry, and limited large-scale clinical data continue to restrict widespread application. Additionally, optimization of photosensitizer pharmacokinetics and improved tumor targeting are critical to achieve consistent therapeutic outcomes.

Future research should focus on developing hybrid platforms that integrate LLLT with nanoparticle-based delivery systems, immune checkpoint inhibitors, and gene therapy to achieve synergistic effects. Advances in multiphoton and femtosecond laser systems could also enable deeper, highly localized activation with minimal collateral damage. Comprehensive clinical trials and dosimetric standardization are essential to translate preclinical findings into effective clinical protocols. In summary, LLLT-based therapies hold transformative potential for breast cancer management, bridging the gap between conventional cytotoxic therapies and next-generation, light-guided molecular medicine. Continued interdisciplinary efforts combining laser physics, nanotechnology, and oncology are expected to define the next frontier of precision phototherapy.

## Data Availability

All data are available in the manuscript and supplementary files.
